# Demographic, clinical and genetic characteristics of patients with amyotrophic lateral sclerosis from two specialised centres in Austria

**DOI:** 10.1007/s00415-025-13614-y

**Published:** 2026-01-10

**Authors:** Omar Keritam, Vera Elisabeth Kleinveld, Sigrid Klotz, Haluk Caliskan, Marita Mayerhofer, Merve Sener, Fiona Jäger, Rosa Weng, Daniel Bormann, Isabel Pugna, Johannes Gebert, Ivan Fedak, Andreas Renner, Lukasz Antoniewicz, Jakob Rath, Gudrun Zulehner, Martin Krenn, Fritz Zimprich, Wolfgang N. Löscher, Hakan Cetin

**Affiliations:** 1 Department of Neurology, Medical University of Vienna, Waehringer Guertel 18-20, 1090 Vienna, Austria; 2 Comprehensive Centre for Clinical Neurosciences and Mental Health, Medical University of Vienna, Vienna, Austria; 3 Department of Neurology, Medical University of Innsbruck, Innsbruck, Austria; 4 Division of Neuropathology and Neurochemistry, Department of Neurology, Medical University of Vienna, Vienna, Austria; 5 Department of Neurology, Clinic Ottakring, Vienna, Austria; 6 Division of Pulmonology, Department of Internal Medicine II, Medical University of Vienna, Vienna, Austria; 7 Comprehensive Center for Chest Diseases, Medical University of Vienna, Vienna, Austria

**Keywords:** Motor neuron disease, Amyotrophic lateral sclerosis, Primary lateral sclerosis, Progressive muscular atrophy

## Abstract

**Background:**

Amyotrophic lateral sclerosis (ALS) is a neurodegenerative disorder characterised by progressive muscle weakness and ultimately death from respiratory failure. Heterogeneity in disease trajectories and outcomes among patients with ALS (pwALS) is influenced by healthcare access, rehabilitation, and palliative care, but real-world data on demographic and clinical characteristics remain scarce in many countries, including Austria.

**Objectives:**

To characterise the demographic, clinical, and genetic landscape of pwALS in Austria.

**Methods:**

In this retrospective cohort study, we included pwALS diagnosed according to the Gold Coast criteria and treated at two large tertiary referral centres. Demographic, clinical, and genetic data were extracted from the local ALS registries, and survival was determined via linkage with *Statistik Austria*, censored in December 2023.

**Results:**

A total of 341 patients with motor neuron disease were included (44.9% female), of whom 5% were diagnosed with primary lateral sclerosis and 2.9% with progressive muscular atrophy. Among pwALS (*n* = 314), spinal onset was most common (67.2%), followed by bulbar onset (29.6%) and respiratory onset (2.5%). Median survival from symptom onset was 36.0 months (IQR 20.0–74.0), with age at onset (HR 1.04, 95% CI 1.02–1.05; *p* < 0.0001), diagnostic delay (HR 0.97, 95% CI 0.96–0.98; *p* < 0.0001), and PEG tube placement (HR 0.72, 95% CI 0.50–1.00; *p* = 0.0478) as the only independent predictors of survival. (Likely) pathogenic variants were identified in 5.5% of patients, including two in *SOD1* and one each in *C9orf72, OPTN*, *TARDBP,* and *FUS*.

**Conclusions:**

This study provides the first comprehensive description of the demographic, clinical, and genetic characteristics of pwALS in Austria, offering valuable real-world insight into disease presentation and genetic diversity.

**Supplementary Information:**

The online version contains supplementary material available at 10.1007/s00415-025-13614-y.

## Introduction

Amyotrophic lateral sclerosis (ALS) is a neurodegenerative disease characterised by progressive loss of motor neurons, resulting in limb muscle and bulbar weakness, respiratory insufficiency, and death within a few years from symptom onset [1]. The disease is clinically heterogeneous, with substantial variation in site of onset, pattern of symptom propagation, and relative involvement of upper and lower motor neurons. Distinct phenotypes such as flail arm or flail leg syndrome predominantly affecting lower motor neurons in the limbs, differ substantially in prognosis from ALS with bulbar or respiratory onset. At the extremes of the clinical spectrum, primary lateral sclerosis (PLS) and progressive muscular atrophy (PMA) mainly involve upper or lower motor neurons, respectively, and are associated with longer survival [2, 3]. In addition to these biological differences, variations in healthcare infrastructure and the organisation of ALS care could further contribute to the heterogeneity observed in patient outcomes. These differences may include access to palliative care, rehabilitation services, and respiratory support, all of which influence outcome. Despite the importance of these factors, real-world data capturing the demographic, clinical, genetic, and treatment characteristics of patients with ALS (pwALS) remain scarce in many countries, including Austria. However, understanding these differences is critical to optimising care delivery and tailoring interventions to local needs.

To address this gap in Austria, we conducted a retrospective cohort study involving two major tertiary ALS centres in eastern and western Austria, and comprehensively analysed demographic, clinical, biomarker, genetic, and treatment data from ALS patients.

## Methods

### Study design and patient ascertainment

In this retrospective cohort study, registries with systematically collected data sets at the Departments of Neurology of the Medical Universities of Vienna and Innsbruck were utilised, covering time periods between January 2009 and July 2023 in Vienna and between June 2019 and July 2023 in Innsbruck. All patients with ALS, PMA or PLS were included if they met the Gold Coast diagnostic criteria or the consensus criteria for the diagnosis of PLS [4, 5], which were retrospectively assessed using available clinical records. The Gold Coast criteria, specifically developed to increase diagnostic sensitivity compared with earlier criteria [4, 6], were applied to minimise the risk of under-ascertainment and to ensure that the study cohort is representative of ALS according to contemporary diagnostic standards. Other monogenetic motor neuron diseases, specifically spinal muscular atrophy and spinal and bulbar muscular atrophy (Kennedy’s disease), were not considered for this study. Survival status was determined via data linkage with *Statistik Austria*, with follow-up data censored in December 2023.

### Data collection

Clinical, demographic, and genetic data were extracted from local ALS registries and electronic health records. Collected clinical parameters included age at onset, motor neuron disease subtype, overlap with frontotemporal dementia [7, 8], site of onset, diagnostic delay, family history, genetic testing, treatments (riluzole, non-invasive ventilation (NIV), percutaneous endoscopic gastrostomy (PEG), and ALS Functional Rating Scale-Revised (ALSFRS-R) scores. ALSFRS-R scores were collected systematically from 2018 onwards, whereas earlier data were available only sporadically. Disease progression rate was calculated using the standard formula ((48–last ALSFRS-R score)/months from onset to last assessment) and categorised as fast (> 1 point/month), intermediate (0.5–1 point/month), or slow (≤ 0.5 points/month) [9–13]. During the study period, i.e., prior to the approval of Tofersen by the European Medicines Agency, genetic testing was preferentially offered to patients aged < 50 years and to those with a positive family history. Targeted single gene testing was performed using Sanger sequencing, repeat-primed PCR, or multiplex ligation-dependent probe amplification. Whole-exome and gene panel sequencing were performed for broader analyses of ALS-associated genes, depending on the laboratory and time period. Repeat expansions in C9orf72 were assessed with a cutoff of 61 repeats. All genes tested in our cohort are summarised in Supplementary Table 1. Only pathogenic or likely pathogenic variants according to the standards of the American College of Medical Genetics and Genomics (ACMG) were reported [14]. Serum neurofilament light chain (NfL) was measured in selected patients as part of the diagnostic work-up to support clinical diagnosis and to aid in prognostic assessment.

### Statistical analyses

Descriptive analyses were performed using means with standard deviations (SD) or medians with interquartile range (IQR), as appropriate. Descriptive data were compared based on treatment centre using the Mann–Whitney test or the Fisher’s exact test. Kaplan–Meier survival curves with log-rank tests were used to compare survival among classical ALS, PLS and PMA subgroups, within the ALS subgroup between patients with spinal onset vs. bulbar/respiratory onset, and between disease progression rates. Cox proportional hazard regression models assessed the effects of sex, age at onset, diagnostic delay, site of onset, riluzole treatment, NIV, and PEG on survival without invasive ventilation in the ALS subgroup. Significant variables in univariate analyses were included in multivariate regression models. Hazard ratios (HR) and 95% confidence intervals (95% CI) were reported. Spearman correlation was performed post-hoc to assess the relationship between diagnostic delay and disease progression rate. Group comparisons of NfL levels between subgroups were performed using unpaired *t* tests or one-way ANOVA with Tukey’s post hoc comparison tests, and means with standard errors (SEM) were reported. A two-sided *p* value of < 0.05 was considered statistically significant. Statistical analysis and graphical illustration were performed using GraphPad Prism version 10.6.0 for macOS (GraphPad Software, Boston MA, USA).

## Results

### Demographic and clinical characteristics

A total of 341 patients were included, with 261 patients (76.5%) treated in Vienna and 80 patients (23.5%) in Innsbruck (Table 1). Female patients accounted for 44.9% of the whole cohort. Most patients (92.1%) were diagnosed with ALS, while 5.0% had PLS and 2.9% PMA. Median age at onset was 63.0 years (IQR 53.0–70.0) in the ALS subgroup, and 60.0 years in both PLS and PMA (IQRs 49.0–67.0 and 55.5–68.3, respectively). Median diagnostic delay in pwALS was 9.0 months (6.0–15.0). Within the ALS subgroup, spinal onset was most common (67.2%), followed by bulbar onset (29.6%) and respiratory onset (2.5%). ALSFRS-R scores were available in 64.0% of pwALS, allowing classification of disease progression as fast (38.3%), intermediate (33.8%), or slow (27.9%). In addition, 6 patients (1.9%) suffered from a concomitant frontotemporal dementia (FTD) and family history (ALS or FTD) was positive in 35 patients (10.3%). In the ALS subgroup, 246 patients (78.3%) received riluzole, while a percutaneous endoscopic gastrotomy (PEG) tube was implanted in only 20.7%. Non-invasive ventilation (NIV) and invasive ventilation (IV) were used in 9.6% and 1.6% of pwALS, respectively. Patients treated in Vienna received a PEG tube or NIV more frequently than those treated in Innsbruck before loss to follow-up (PEG: 24.4% vs. 8.3%, *p* = 0.0017; NIV: 11.6% vs. 2.8%, *p* = 0.0220). No other significant differences in the descriptive data were found between patients treated in Vienna and patients treated in Innsbruck.

**Table 1:** Demographic and clinical characteristics

	Whole cohort, *n* = 341
Sex	
Female	153 (44.9%)
Male	188 (55.1%)
Motor neuron disease subtype	
PLS	17 (5.0%)
PMA	10 (2.9%)
ALS	314 (92.1%)
Onset type	
Spinal	211 (67.2%)
Bulbar	93 (29.6%)
Respiratory	8 (2.5%)
Unknown	2 (0.6%)
Progression rate*	
Fast progressors	77 (24.5%)
Intermediate	68 (21.7%)
Slow	56 (17.8%)
Unknown	113 (36.0%)
*Median point loss in ALSFRS-R per month (IQR)	0.8 (0.5–1.4)
ALS/FTD overlap	6 (1.9%)
Median age at onset, years (IQR)	
ALS	63.0 (53.0–70.0)
PLS	60.0 (49.0–67.0)
PMA	60.0 (55.5–68.3)
Median diagnostic delay in ALS, months (IQR)	9.0 (6.0–15.0)
Family history	
ALS or dementia	35 (10.3%)
Unknown	18 (5.3%)
Genetic testing, total number of patients	110 (32.3%)
Targeted single gene testing, number of patients	73 (66.4%)
(Likely) pathogenic variant	1 (1.4%)
Gene panel sequencing, number of patients	25 (22.7%)
(Likely) pathogenic variant	2 (8.0%)
Whole exome sequencing, number of patients	61 (55.5%)
(Likely) pathogenic variant	3 (4.9%)
Therapies in ALS	
Riluzole	246 (78.3%)
Unknown	11 (3.5%)
PEG	65 (20.7%)
Unknown	5 (1.6%)
NIV	30 (9.6%)
Unknown	12 (3.8%)
IV	5 (1.6%)
Unknown	13 (4.1%)

For detailed information on genes analysed in single gene and panel sequencing refer to supplementary Table 1. *ALS* Amyotrophic Lateral Sclerosis; *ALSFRS-R* ALS Functional Rating Scale; *FTD* Frontotemporal Dementia; *IQR* Interquartile Range; *IV* Invasive Ventilation; *NIV* Non-Invasive Ventilation; *PEG* Percutaneous Endoscopic Gastrostomy; *PMA* Progressive Muscular Atrophy; *PLS* Primary Lateral Sclerosis

### Survival analyses

**Fig. 1:**
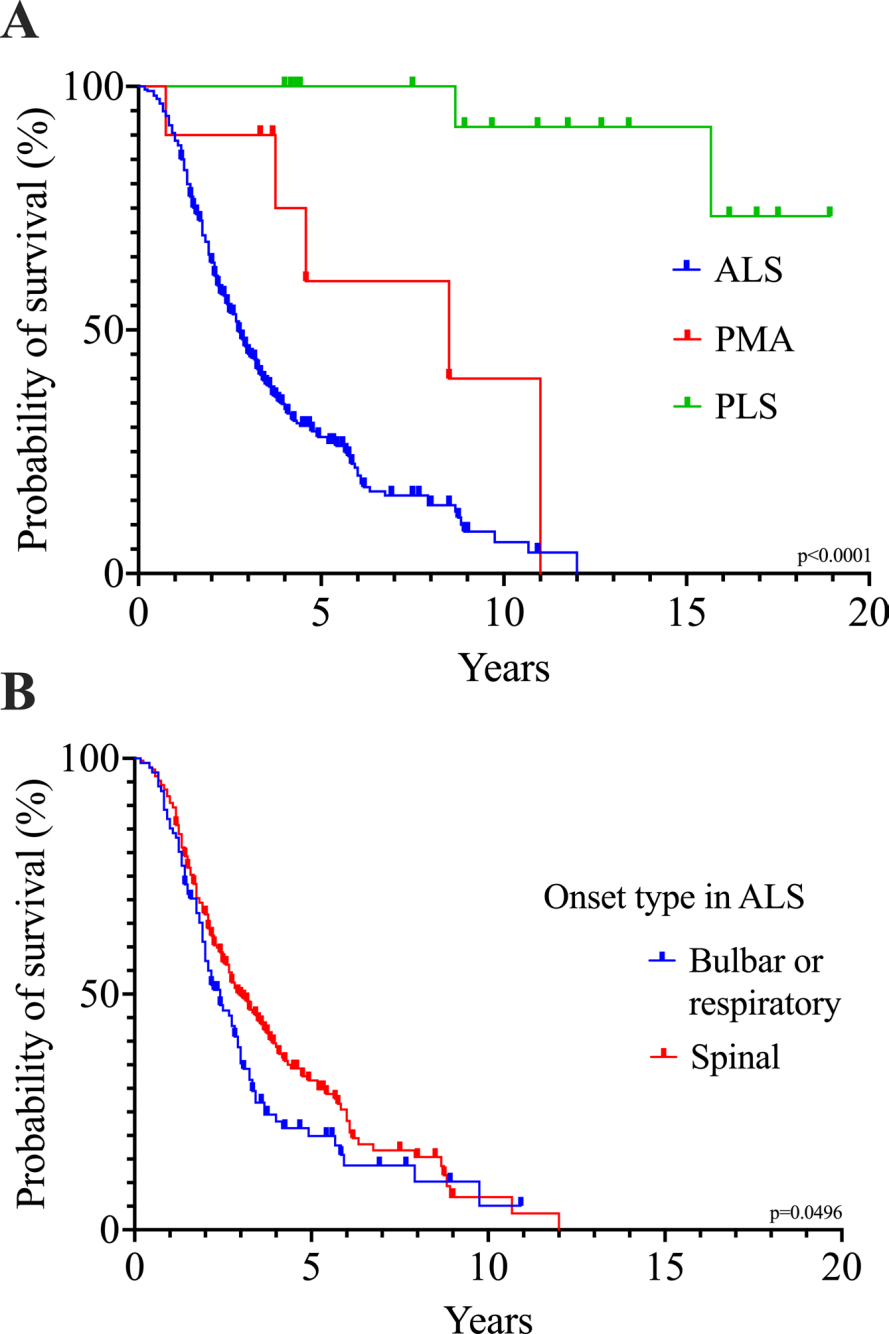
Kaplan–Meier survival analyses revealed significant differences between various subgroups. **A** Survival was shorter in patients with classical ALS (median 33.0 months [= 2.8 years], IQR 19.0–68.0) than in patients with PMA (median 102.0 months [= 8.5 years], IQR 45.0–132.0) or PLS (median not defined). **B** Spinal onset ALS was associated with a longer survival time (median 36.0 months [= 3.0 years], IQR 20.0–72.0) as compared to bulbar or respiratory onset ALS (median 29.0 months [= 2.4 years], IQR 17.0–44.0). *ALS* Amyotrophic Lateral Sclerosis. *IQR* Interquartile Range. *PMA* Progressive Muscular Atrophy. *PLS* Primary Lateral Sclerosis

Median survival time from symptom onset in the whole cohort was 36.0 months (IQR 20.0–74.0). Patients with ALS had significantly shorter survival (median 33.0 months, IQR 19.0–68.0) compared to those with PMA (median 102.0 months, IQR 45.0–132.0) or PLS (median not defined; *p* < 0.0001; Fig. 1A). In the PLS subgroup, the median time until censoring was 131.0 months (IQR 53.0–194.0) in 15 patients (88.2%), while only two patients deceased 104 and 188 months after symptom onset, respectively. Among patients with ALS, survival differed by region of onset. Patients with spinal onset survived longer (median 36.0 months, IQR 20.0–72.0) than those with bulbar or respiratory onset (median 29.0 months, IQR 17.0–44.0; *p* = 0.0496; Fig. 1B). Survival was also strongly associated with the rate of disease progression. Patients with fast progression had the shortest survival (median 20.0 months, IQR 15.0–31.0), those with slow progression the longest (median 95.0 months, IQR 57.0–117.0), while patients with intermediate progression had a median survival of 42.0 months (IQR 26.0–68.0; *p* < 0.0001). In univariate analyses, older age at onset (HR 1.03, 95% CI 1.02–1.04; *p* < 0.0001) and bulbar onset (HR 1.33, 95% CI 1.00–1.74; *p* = 0.0442) were associated with poorer prognosis, whereas diagnostic delay (HR 0.97, 95% CI 0.96–0.98; *p* < 0.0001) and PEG tube placement (HR 0.68, 95% CI 0.48–0.94; *p* = 0.0196) predicted longer survival time. Sex, treatment with riluzole, and NIV were not significantly associated with survival. In the multivariate analysis, only age at onset (HR 1.04, 95% CI 1.02–1.05; *p* < 0.0001), diagnostic delay (HR 0.97, 95% CI 0.96–0.98; *p* < 0.0001) and PEG tube insertion (HR 0.72, 95% CI 0.50–1.00; *p* = 0.0478) remained independent predictors of survival. A post-hoc analysis revealed a moderate, but highly significant negative correlation between time from disease onset to diagnosis and disease progression rate (Spearman r =  − 0.4798; *p* < 0.0001).

### Neurofilaments

**Fig. 2:**
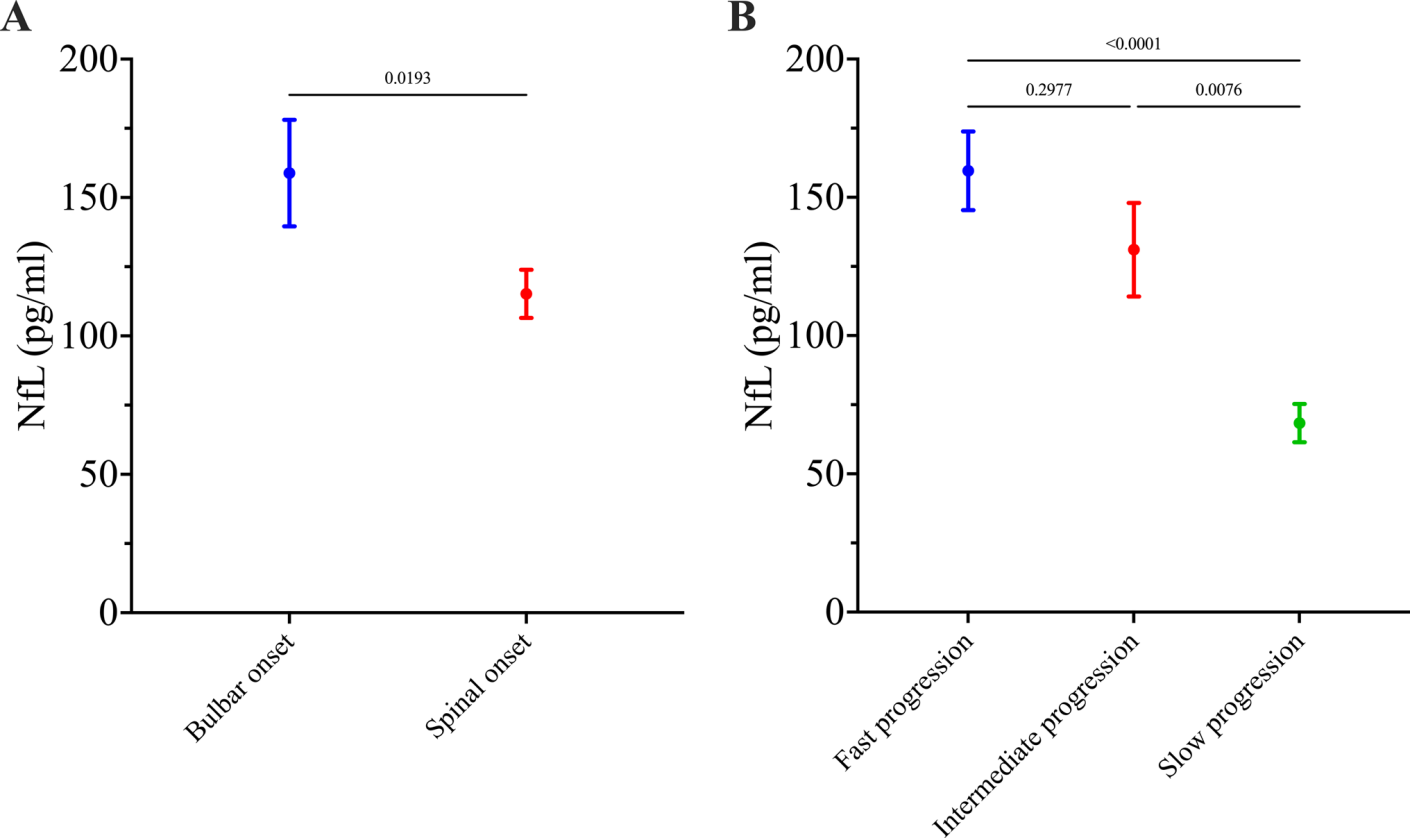
**A** Mean NfL concentration was significantly higher in patients with bulbar onset ALS (*n* = 29, mean 159 pg/ml, SEM 19) than in patients with spinal onset ALS (*n* = 71, mean 115 pg/ml, SEM 9). **B** Patients with a slow progression rate had a significantly lower mean NfL concentration (*n* = 27, mean 68 pg/ml, SEM 7) than patients with an intermediate (*n* = 30, mean 131 pg/ml, SEM 17) or with a fast progression rate (*n* = 35, mean 160 pg/ml, SEM 14), and NfL concentrations were not significantly different between patients with fast and intermediate progression rates. *p* values are included in the graphs. *ALS* Amyotrophic Lateral Sclerosis. *NfL* Neurofilament Light Chain. *SEM* Standard Error

NfL levels were measured in 105 ALS patients (33.4%) at a median time from symptom onset of 11.0 months (IQR 6.0–21.0), with a mean concentration of 128 pg/ml (SEM $\pm$ 8). NfL concentrations were significantly higher in patients with bulbar onset ALS (*n* = 29, mean 159 pg/ml, SEM $\pm$ 19) compared to those with spinal onset (*n* = 71, mean 115 pg/ml, SEM $\pm$ 9; *p* = 0.0193; Fig. 2A). One-way ANOVA revealed significant differences in NfL levels (*p* < 0.0001) across patient subgroups defined by disease progression rate (Fig. 2B). NfL concentrations were significantly higher in patients with fast (*n* = 35, mean 160 pg/ml, SEM $\pm$ 14) or intermediate (*n* = 30, mean 131 pg/ml, SEM $\pm$ 17) progression than in patients with slow progression (*n* = 27, mean 68 pg/ml, SEM $\pm$ 7; *p* < 0.0001 and *p* = 0.0076, respectively), but did not differ significantly between the subgroups with fast and intermediate progression.

### Genetic findings

Genetic testing was performed in a total of 110 patients (32.3%), of whom 45.5% were female and 15.5% had a positive family history. In this subgroup, median age at onset was 58.0 years (IQR 47.0–67.0). Single gene sequencing, gene panel sequencing and whole exome sequencing were performed in 66.4%, 22.7% and 55.5%, respectively (Table 1). In 48 patients (43.6%) multiple methods for genetic testing were utilised. A total of 6 (likely) pathogenic variants (5.5%) were identified. In a patient with a positive family history for *FUS*-related ALS, a pathogenic variant was detected by single gene testing in the *FUS* gene (NM_004960.4: c.1562G > A, p.Arg521His). In addition, two pathogenic variants were detected by panel sequencing in *TARDBP* (NM_007375.4: c.943G > A, p.Ala315Thr) and *C9orf72* (NM_001256054.1: c.−45 + 163GGGGCC). Whole-exome sequencing revealed an *SOD1* variant (NM_000454.4:c.435G > C, p.Leu145Phe) in two patients and an *OPTN* variant (NM_001008211.1:c.1078_1079delAA, p.Lys360ValfsTer18) in one patient (Table 2).

**Table 2:** Pathogenic and likely pathogenic genetic variants, according to ACMG criteria  [14]

Gene	Variant	*n* = 110
*SOD1*	NM_000454.4:c.435G > C, p.Leu145Phe (heterozygous)	2 (1.8%)
*OPTN*	NM_001008211.1:c.1078_1079delAA, p.Lys360ValfsTer18 (homozygous)	1 (0.9%)
*TARDBP*	NM_007375.4: c.943G > A, p.Ala315Thr (heterozygous)	1 (0.9%)
*FUS*	NM_004960.4: c.1562G > A, p.Arg521His (heterozygous)	1 (0.9%)
*C9orf72*	NM_001256054.1: c.−45 + 163GGGGCC	1 (0.9%)

*TARDBP* variant and *C9orf72* repeat expansion were found in a gene panel analysis. *FUS* variant was found in a single gene analysis. *SOD1* and *OPTN* variants were found in WES. *ACMG* American College of Medical Genetics and Genomics; *WES* Whole-exome sequencing

## Discussion

In this study, we characterised a cohort of pwALS treated at two major tertiary referral centres in Vienna and Innsbruck, representing two large, specialised ALS clinics in Austria. This setting provides valuable real-world insight into the clinical, demographic, and genetic landscape of ALS in Austria.

The clinical and demographic characteristics observed in our cohort largely correspond to previously published data, including the distribution of motor phenotypes, age at onset, diagnostic delay, and survival time [15–23]. Notably, the proportion of patients with an ALS/FTD overlap syndrome was comparatively low [24–27], as neuropsychological testing was only performed in selected patients at our clinics, which likely led to underestimation. The survival analyses confirmed well-established prognostic markers. Patients with spinal onset and slower ALSFRS-R progression had longer survival, whereas older age at onset was associated with poorer outcome. A longer diagnostic delay was also linked to extended survival, likely reflecting that patients with a slower disease progression were referred to our centres later than those patients with fast disease progression. This interpretation is supported by the moderate but highly significant negative correlation between diagnostic delay and ALSFRS-R decline. These results are consistent with pervious large multicentre studies and meta-analyses [28, 29]. In our multivariate analysis, PEG tube placement, but not NIV, was associated with improved survival. However, the available data did not allow assessment of adherence to consensus recommendations or the precise timing of these interventions, many of which were performed outside the study centres. Our results should thus be interpreted with caution, as these potentially confounding factors may have influenced our analyses. Importantly, PEG or NIV use typically reflects advanced disease stage, and as we were not able to stratify analyses by disease stage or by the exact timing of these interventions, the observed effects (or lack thereof) cannot be generalised to the whole cohort. Early initiation of PEG and NIV has been shown to prolong survival in pwALS [30–33], and the limited use of these interventions in our cohort may reflect restricted access to structured multidisciplinary care during the study period, which has also been shown to improve outcome in pwALS [34].

Riluzole treatment was not associated with prolonged survival in our cohort, which is consistent with previous prospective and retrospective studies reporting a survival advantage limited to the early treatment period, with convergence and crossing of survival curves after 6–26 months [35–38]. In an Austrian population-based study, the protective effect of riluzole diminished after a period of 6 months after treatment initiation [35]. More recent multicentre data from the PRECISION–ALS database suggested a survival advantage in patients treated with riluzole during a 10-year follow-up period [39]. However, this effect was strongly influenced by disease progression rate, with the greatest advantage observed in patients with fast-progressing ALS. Unfortunately, we were not able to analyse riluzole effects in our cohort due to low small case numbers.

NfL levels differed significantly between patients with different disease phenotypes, with higher concentrations measured in patients with bulbar onset compared with spinal onset. These findings are in line with previous studies [40], although mean NfL concentrations were higher in our cohort (spinal-onset ALS: 115.2 vs. 64.1 pg/ml in [40]; bulbar-onset ALS: 158.8 vs. 92.7 pg/ml in [40]). One possible explanation for this difference might be the faster median ALSFRS-R progression rate observed in our cohort (0.8 vs. 0.5 in [40]). Notably, faster disease progression was associated with higher NfL concentrations both in this study as well as in previously published studies [10, 13, 41–43]. Our results thereby confirm the established association between elevated NfL levels and accelerated neurodegeneration in ALS.

Genetic analyses in our cohort revealed (likely) pathogenic variants in 5.5% of tested patients, including the *SOD1*, *C9orf72*, *FUS*, *TARDBP*, and *OPTN* genes, which is lower than commonly reported in the literature [44–48]. One possible explanation is that only 56% of tested patients underwent whole-exome sequencing, which has been shown to increase detection rates [49]. In addition, the median age at onset in the genetically tested subgroup was 58 years and thus relatively high, and only half of the patients with a positive family history underwent testing, which might have further reduced the probability of detecting pathogenic variants in our cohort.

Several limitations of this study must be acknowledged. First, the retrospective design may have introduced information bias. Limited information on the timing of initiation and adherence to treatment with riluzole, PEG, and NIV precludes robust interpretation of their effects on survival. In addition, the proportions of patients with an ALS/FTD overlap syndrome or with underlying causative variants in our cohort are likely underestimated, as neuropsychological and genetic testing were only performed in selected patients. ALS/FTD was diagnosed based on clinical assessments documented in the medical records, in line with established diagnostic concepts for FTD in ALS, but without systematic neuropsychological testing, which likely contributed to the lower prevalence observed in our cohort as compared with international studies [24–27]. Finally, although we estimate that patients treated at the Medical Universities of Vienna and Innsbruck may constitute a large proportion of the total ALS cohort in Austria [35], the inclusion of patients from only two tertiary centres limits the generalisability of our results. As there are no specific healthcare policies for the management of pwALS in Austria, care for these patients varies substantially between hospitals, particularly between secondary and tertiary care centres. Differences between the Medical Universities of Vienna and Innsbruck may also have influenced the demographic and clinical data reported in this study. For example, end-stage patients in Vienna were often managed by general practitioners or community-based neurologists, as no structured palliative care setting for ALS patients existed at the time, whereas in Innsbruck such patients were transferred earlier to palliative care specialists, potentially affecting access to interventions, such as ventilation or PEG. Access to invasive home ventilation is also limited by resources, as public healthcare support typically covers only part of the associated costs. Importantly, the establishment of national databases and standardised care policies could help minimise these differences, ensuring more equitable access to genetic testing, clinical interventions, and end-of-life care. This would improve consistency of clinical management and enable a more reliable characterisation of the demographic, clinical and genetic landscape, which is pivotal given expected treatment advances over the coming decades.

In summary, we present the first comprehensive demographic, clinical, and genetic characterisation of an Austrian ALS cohort. Patients treated at the two largest ALS centres in Austria offer valuable insight into the spectrum of ALS care and phenotype distribution in the country. Our results largely align with previously published data, but subtle differences, particularly in the use of supportive interventions and the frequency of genetic findings, warrant further investigation. Future prospective studies including nationwide data and systematic genetic testing as recently included at both centres may help to refine the understanding of ALS in Austria.

## Supplementary Information

Below is the link to the electronic supplementary material.

Supplementary file1 (DOCX 25 KB)

## Data Availability

No patient data or study-related documents are shared within this paper. Reasonable requests from qualified investigators will be considered by the corresponding author in accordance with applicable privacy regulations.
