# Supplementary material for: Demographic, clinical and genetic characteristics of patients with amyotrophic lateral sclerosis from two specialised centres in Austria
Source: J Neurol. 2026 Jan 10;273(1):74. doi: 10.1007/s00415-025-13614-y (PMC12789124; doi:10.1007/s00415-025-13614-y)
Supplement: Supplementary file 1 — Supplementary file1 (DOCX 25 KB) [file 415_2025_13614_MOESM1_ESM.docx]

**Supplementary Table 1** Detailed information on all genes tested in targeted single gene testing or panel sequencing in our cohort.

|  | **Tested cohort, n=110** |
| --- | --- |
| Targeted single gene testing, number of patients | 73 (66.4%) |
| Genes analysed |  |
| *C9orf72* | 50 (68.5%) |
| *SOD1* | 15 (20.5%) |
| *AR* | 10 (13.7%) |
| *FUS* | 5 (6.8%) |
| *SMN1* | 5 (6.8%) |
| *SLC52A2/A3* | 1 (1.4%) |
| *TARDBP* | 1 (1.4%) |
| *FXN* | 1 (1.4%) |
| *NOTCH3* | 1 (1.4%) |
| *KIF5A* | 1 (1.4%) |
| *ATM* | 1 (1.4%) |
| *POLG* | 1 (1.4%) |
| *MAPT* | 1 (1.4%) |
| *ATXN3* | 1 (1.4%) |
| Gene panel sequencing, number of patients | 25 (22.7%) |
| Genes included in panels |  |
| Panel for amyotrophic lateral sclerosis | 23 (92.0%) |
| *ALS2, ANG, ANXA11, AR, ASCC1, ATL1, ATXN2, BSCL2, C9orf72, CHCHD10, CHMP2B, DCTN1, DNAJB2, FIG4, FUS, GBE1, GRN, HEXA, HEXB, HNRNPA1, KIF5A, LRP12, MATR3, NEK1, NOP56, OPTN, PFN1, REEP1, SETX, SIGMAR1, SLC52A2, SLC52A3, SMN1, SOD1, SPART, SPAST, SPG11, SPG7, SPTLC1, TARDBP, TBK1, TUBA4A, UBQLN2, VAPB, VCP* |  |
| Panel for hereditary spastic paraplegia | 2 (8.0%) |
| *L1CAM, PLP1, ATL1, SPG4, CYP7B1, SPG7, KIAA0196, KIF5A, SPG11, RTN2, HSPD1, ZFYVE26, BSCL2, ERLIN2, SPG20, SPG21, SLC16A2, B4GALNT1, DDHD1, KIF1A, REEP1, ZFYVE27, FA2H, PNPLA6, SLC33A1, C19orf12, NT5C2, GBA2, AP4B1, AP5Z1, TECPR2, AP4M1, AP4E1, AP4S1, VPS37A, DDHD2, C12orf65, CYP2U1, TFG, ARL6IP1, AMPD2, ENTPD1, ALS2, CCT5* |  |
| Panel for spinocerebellar ataxia | 1 (4.0%) |
| *SCA1, SCA2, SCA3, SCA6, SCA8, SCA10, SCA12, SCA17, ATN1, JPH3* |  |
